# Raman Spectroscopy for In-Line Water Quality Monitoring — Instrumentation and Potential

**DOI:** 10.3390/s140917275

**Published:** 2014-09-16

**Authors:** Zhiyun Li, M. Jamal Deen, Shiva Kumar, P. Ravi Selvaganapathy

**Affiliations:** 1 School of Biomedical Engineering, McMaster University, Hamilton, ON L8S 4K1, Canada; E-Mails: liz27@mcmaster.ca (Z.L.); selvaga@mcmaster.ca (P.R.S.); 2 Electrical and Computer Engineering, McMaster University, Hamilton, ON L8S 4K1 Canada; E-Mail: kumars@mcmaster.ca; 3 Mechanical Engineering, McMaster University, Hamilton, ON L8S 4K1, Canada; 4 Electronic and Computer Engineering, Hong Kong University of Science and Technology, Clear Water Bay, Kowloon, Hong Kong, China

**Keywords:** Raman spectroscopy, water monitoring, surface enhanced Raman scattering

## Abstract

Worldwide, the access to safe drinking water is a huge problem. In fact, the number of persons without safe drinking water is increasing, even though it is an essential ingredient for human health and development. The enormity of the problem also makes it a critical environmental and public health issue. Therefore, there is a critical need for easy-to-use, compact and sensitive techniques for water quality monitoring. Raman spectroscopy has been a very powerful technique to characterize chemical composition and has been applied to many areas, including chemistry, food, material science or pharmaceuticals. The development of advanced Raman techniques and improvements in instrumentation, has significantly improved the performance of modern Raman spectrometers so that it can now be used for detection of low concentrations of chemicals such as in-line monitoring of chemical and pharmaceutical contaminants in water. This paper briefly introduces the fundamentals of Raman spectroscopy, reviews the development of Raman instrumentations and discusses advanced and potential Raman techniques for in-line water quality monitoring.

## 1. Introduction

Water is essential to our health and well-being, and it is also crucial to the sustainable development of human society. However, the quality of drinkable water worldwide is under severe stress due to a wide range of natural and human activities, including the discharge of toxic chemicals from industry, nitrate and pesticide contamination in agriculture, and long distance atmospheric transport of pollutants [1]. For purpose of water safety, the monitoring of water contaminants is of great importance. The US Environmental Protection Agency (EPA) has set the maximum contamination level (MCL) for most of the contaminants in drinking water, including the microorganisms, disinfectants, disinfection by-products, inorganic chemicals, organic chemicals and radionuclides. The MCL determines the limit of detection of most water monitoring techniques.

Water quality can be characterized by concentrations of microbiological, organic and inorganic contaminants, or by some of the physical characteristics including pH, temperature or turbidity [2]. Various commercial instruments have been used to detect different water quality indicators. For example, a TOC (total organic carbon) analyzer is used for total organic carbon measurement. Gas chromatography and enzyme-based systems are able to detect chemical contaminants and immunoassays, respectively. Flow cytometry has been used for the detection of microbial contaminants. A detailed review of different detection systems can be found in [3].

Most of the analyses methods described above are laboratory based and require skilled personnel for their operation. Therefore, they are expensive to perform and require transportation of the sample to the laboratory. These factors result in a delay in response to contamination events, which is detrimental to water safety and public health. Thus, there is a growing and urgent need for a robust, low-cost, continuous, fast and accurate on-site detection system.

In the past decades, high speed detection systems for in-line water monitoring have attracted lots of interest in industry and academia, and many advanced water detection systems are commercially available to measure various water quality indicators [3]. Among them, vibrational spectroscopy has been extensively studied because of its non-contact, non-invasive sampling, minimum sample preparation and rapid detection of chemical and microbial contaminants, which are the most common contaminants in water [2,4]. Table 1 lists some of the water contaminants that can be measured by the vibrational spectroscopy and the MCL set by EPA [5].

Vibrational spectroscopy refers to measurement of the vibrational energy levels in a molecule which are associated with its chemical bonds. The vibrational spectrum is rich in chemical composition information of the sample, so it has been applied for chemical analysis, such as water quality, material science and probing physical states. Infrared (IR) and Raman Spectroscopy are the two most commonly used vibration spectroscopy techniques for chemical and biological analysis. Within IR spectroscopy, the middle infrared (MIR 2.5–16 μm) spectroscopy is the most developed technique for molecule structure analysis, since most fundamental transitions occur at MIR. However, due to the strong light absorption of water in MIR, special care is usually taken when measuring liquid samples. For instance, the thickness of sample holder for liquid samples is usually in the range of micrometers to ensure that a strong enough signal is obtained. Alternatively, attenuated total reflection (ATR) method is used to strengthen the interaction between the IR signal and liquid sample [6]. Finally, some pre-concentration techniques have also been used to enhance the absorption signal [7].

The near infrared (NIR) spectroscopy covers the transition from 780 to 2526 nm. Different from the MIR spectroscopy, the NIR spectroscopy originates from the overtones and combinations of fundamental vibrations [8]. Since water absorption in the NIR is weaker than in the MIR, NIR is more suitable for direct measurement of liquid samples. However, the overlapping and weak intensity of the absorption bands make the NIR spectrum quite difficult to analyze, and multivariate techniques such as principal component analysis and partial least squares regression [2] are used for NIR spectra analysis. Although NIR spectroscopy is not as well-established as MIR spectroscopy, it is increasingly considered as a promising technique to detect inorganic materials [9,10].

Since water is a weak Raman scatterer, Raman spectroscopy is superior to other vibrational spectroscopies particularly in some applications such as biomedical diagnosis, tissue imaging [11–13] and monitoring of liquid samples. Raman spectroscopy was first used in 1970 to detect organic contaminants in water [14]. However, because of the lower Raman scattering efficiency, strong fluorescence interference and lack of sensitive instruments, it was not widely investigated before the 1980s. With the development of high detection efficiency, low noise, silicon based detector arrays, stable and high power laser diodes, high throughput and high resolution spectrometers, Raman spectroscopy has achieved tremendous progress since the late 1980s [15]. In addition to improved hardware, the advent of advanced Raman techniques such as surface enhanced Raman spectroscopy, the limit of detection has been dramatically improved thus making this technique suitable for monitoring chemical contaminants in water.

In this paper, we introduce the basic principle of Raman spectroscopy. Then, a detailed review of the development of its instrumentation is given. Next, the most promising Raman techniques for the detection of water contaminants are discussed. Finally, conclusions and future trends in Raman spectroscopy are provided.

## 2. Raman Spectroscopy

### 2.1. Scattering

Raman scattering originates from the interaction between incident electromagnetic radiation and molecular vibration. According to classical theory, when a molecule is illuminated or placed in an electric field *E* defined as *E* = *E*_0_ cos 2 πν_0_*t*, an electric dipole moment *P* is induced,

(1) P=αE

α is the polarizability. Considering only lower order Raman scattering, then since molecular vibrations are composed of several normal modes, it can be written as,

(2) $$\alpha =\alpha_{0}+{\left(\frac{\partial \alpha }{\partial Q_{i}}\right)}_{0}Q_{i}+...$$

where *Q* is the normal coordinates of the various vibration modes and can be expressed as, *Q_i_* = *Q_i_*_0_ cos 2 πν*_i_t*. Substituting Equation (2) into the dipole moment *P* gives

(3) $$P=\alpha_{0}E_{0}\cos2{\pi \nu }_{0}t+\frac{1}{2}{\left(\frac{\partial \alpha }{\partial Q_{i}}\right)}_{0}Q_{i0}E_{0}\left[\cos2\pi t\left(\nu_{0}+\nu_{i}\right)+\cos2\pi t\left(\nu_{0}-\nu_{i}\right)\right]+...$$

This dipole moment leads to scattering of the incident light. The first term in Equation (3) with the same frequency as the incident light is usually called *Rayleigh* scattering. The rest of the terms in Equation (3) have frequencies above and below the original frequency (ν_0_ + ν*_i_*, ν_0_ − ν*_i_*), and are called *anti-Stokes* and *Stokes* Raman scattering respectively. Since the magnitude of α_0_ is larger than (∂α/∂*Q_i_*)_0_ *Q_i_*_0_, the intensity of the Rayleigh scattering is much stronger than the Stokes and anti-Stokes Raman scattering. For instance, out of every 10^10^ incident photons only one generates a Raman photon [16], which gives rise to a very low Raman scattering efficiency. Furthermore, Raman scattering only occurs when (∂α/∂*Q_i_*)_0_ ≠0, which means that a vibration mode is Raman active only if the polarizability changes during vibration, and this is known as the selection rule of Raman scattering.

**Figure 1.**
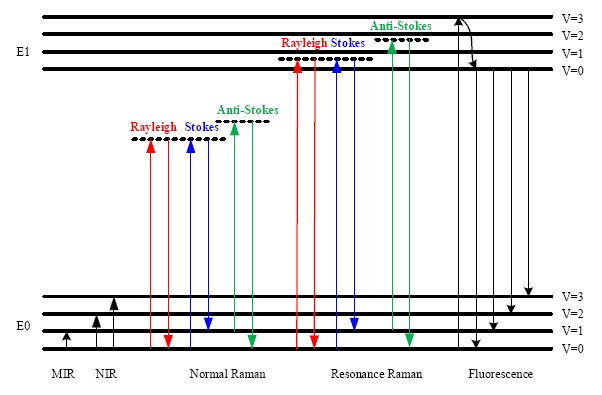
Energy level diagram related to IR absorption, Raman scattering and fluorescence emission.

In Figure 1, the energy level diagram of IR absorption, the scattering and fluorescence processes are shown. For a normal Raman scattering process, a molecule is excited from its ground state (E0) to a virtual state when illuminated by incident radiation such as a laser. Since a virtual state is not a stationary quantized energy level and does not correspond to any eigenvalue, it will decay very fast with the emission of a photon [15]. For Stokes Raman scattering, the initial state of a molecule is the lowest vibrational state of the ground state (E0, *v* = 0). In contrast, the anti-Stokes Raman scattering starts from the higher vibrational energy level (E0, *v* = 1) of the ground state. According to the Maxwell-Boltzmann distribution law, the population of molecules at *v* = 0 is higher than that at *v* = 1, so the Stokes Raman scattering is stronger than the anti-Stokes Raman scattering under normal conditions [17]. Hence, the Stokes spectrum is mainly measured in most commercial Raman spectrometers.

Although the Raman scattering efficiency is very low, if the excitation frequency is close to the frequency of an electronic transition, then scattering enhancements of up to 10^6^ can be achieved [18]. This effect is termed the Resonance Raman scattering, shown in Figure 1. Since the electronic transition frequencies vary among different chemicals, then ideally, a tunable laser is required for Resonance Raman spectroscopy.

The fluorescence emission process is also depicted in Figure 1. In this process, a molecule is excited from the ground state (E0) to one of the vibrational states in the electronic excited state (E1). Through vibrational relaxation, the molecule relaxes back to the lowest excited state by losing excess energy as heat. Then, the molecule transitions from the lowest excited state to one of the vibrational states in the ground state with the emission of a photon. The energy of the emitted photon is lower than that of the incident photon because of the energy loss during vibrational relaxation, and this is also called red shift.

The fluorescence emission spectrum is broad band covering the same wavelength band as the Stokes Raman signal. Since the cross section for fluorescence is larger than for Raman scattering, then detection of Raman scattering is very difficult when strong fluorescence emission is present. To suppress or remove the fluorescence background, various techniques have been used. For instance, it is possible to obtain a fluorescence-free Raman spectrum by using a NIR source. Alternatively, since fluorescence emission is red shifted, the anti-Stokes Raman signal does not overlap with the fluorescence emission. Thus it can be measured and is used in a technique called coherent anti-Stokes Raman spectroscopy (CARS) [19]. Table 2 lists some of the features, instrumentations and applications of the optical processes depicted in Figure 1.

### 2.2. Raman Scattering Intensity

A Raman spectrometer measures the intensity of the Raman signal (Stokes or anti-Stokes) and plots the Raman signal intensity *versus* the frequency shift of the Raman signal relative to the excitation source, which is known as the Raman shift. As can be seen in Figure 1, both Raman shift and IR absorption are related to the fundamental vibrational modes. Since most of the fundamental transitions occur at MIR, MIR sources are mainly used for absorption spectroscopy. Unlike absorption spectroscopy, sources varying from the ultraviolet (UV) to visible or even NIR regions are capable of Raman scattering excitation. The intensity of Raman signal *I_R_* is wavelength dependent and can be expressed as [15,16],

(4) $$I_{R}\propto I_{0}{\left(\nu_{0}\pm \nu_{i}\right)}^{4}N$$

where *N* refers to the number density of molecules, and *I*_0_ is the intensity of the excitation source. The Raman signal intensity *I_R_* in Equation (4) is 4th power dependent on the excitation frequency. To achieve high Raman scattering efficiency, high frequency or short wavelength excitations, such as UV source, are usually preferred. However, most of the modern Raman instruments are equipped with a NIR source or a visible source. UV sources are rarely used due to the unavailability of low cost UV lasers. In addition, the use of UV or even visible sources excitation would induce a fluorescence signal, which is stronger and could potentially overwhelm the Raman signal.

## 3. Instrumentation

**Figure 2.**
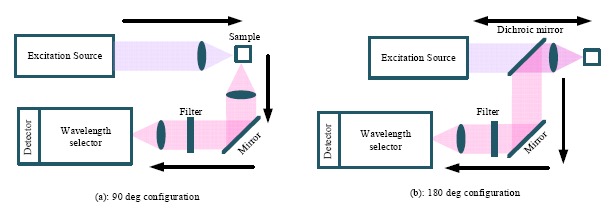
Raman system with: (**a**) 90° and (**b**) 180° configurations.

Instrumentation for the measurement of Raman spectra consists of four components, namely: excitation source; illumination and light collection optics; wavelength selector unit; and the detector. As shown in Figure 2, there are basically two types of light collection systems—90° and 180° configurations [17]. In the 90° degree configuration, the scattering light is collected from the direction perpendicular to the excitation direction. The 90° configuration is easier to setup and is mainly used in the macro-Raman experiments.

In contrast, the scattering signal is collected in the direction opposite to the excitation direction in the 180° configuration, which is also termed back scattering. Additional optics is required in the 180° configuration, such as the use of the dichroic mirror, which reflects the longer wavelength scattering signal and transmits the short wavelength excitation signal. As can be seen in Figure 2, besides the light collection system, the other three major elements in a Raman spectrometer are the excitation source, wavelength selector and detector. These major elements will be discussed in the subsections below.

### 3.1. Excitation Source

The excitation source plays an important role in the performance of a Raman spectrometer, including its sensitivity and stability. Two important parameters of the excitation source are its bandwidth and power. As mentioned above, the frequency shift between the Raman signal and the excitation signal is related to the fundamental vibrational frequency of a molecule. Therefore, a narrow bandwidth or a highly monochromatic beam with high power is preferred for stability and high enough intensity of the Raman spectrum.

Raman spectroscopy was a niche technique in the 1940s and 1950s primarily due to the lack of powerful excitation sources. Initially, a mercury lamp with filters that transmitted a narrow wavelength band was used as the excitation source, but it was very weak. Raman spectroscopy gained mainstream significance with the advent of lasers in the 1960s. A continuous HeNe red wavelength source was first used as a Raman source in 1963 [20]. The development of gas lasers such as Argon and Krypton lasers in the 1970s, drove the application of Raman spectroscopy in the visible and UV regions. In the 1980s, the advent of solid state lasers (YAG laser, 1064 nm) boosted the application of NIR Raman spectroscopy. A typical technique is Fourier Transform (FT) Raman spectroscopy [21,22], by which fluorescence free spectra can be obtained, because the energy of a photon from the NIR region has less probability to excite the molecule from the ground state to the excited electronic state and then emit a fluorescence signal. In many modern continuous wave or time-resolved Raman spectrometers, diode lasers are also used to provide excitation from blue to the NIR regions.

Generally, the excitation source is selected according to the specific application. This is because the excitation bands of most fluorophores are in UV or visible wavelength regions. Therefore, excitation from a longer wavelength, such as in the NIR region, reduces the probability to excite fluorescence signals because of the lower photon energy. Since natural fluorophores exist in most biological samples, then NIR lasers are used in either dispersive or FT-Raman spectrometers for characterization of the biological samples. Furthermore, using lower frequency and power can also avoid damage to the samples. However, since the Raman signal intensity in NIR is relatively low compared to lower wavelength spectrometers, then NIR Raman spectrometers have to be equipped with expensive and sophisticated detectors. In contrast, visible lasers can be used for non-biological applications, such as in nanotechnology or solid state physics. The 532 nm solid state laser and 514 nm ion laser are widely used for measurements of inorganic samples. Advantages of visible excitation are the higher signal-to-noise ratio and better sensitivity. In cases when both fluorescence rejection and high sensitivity are required, a compromise is made between these two factors. The most commonly used source for biological samples is the 785 nm laser, which not only provides some fluorescence rejection, but also works well with the silicon-based detectors [23–25]. In Table 3, commonly used lasers and their applications are provided.

### 3.2. Wavelength Selector

The wavelength selector is the most critical component in a Raman spectrometer, through which the intensity information of individual frequencies is extracted. There are basically two types of wavelength selection mechanisms, dispersive and non-dispersive. A dispersive spectrometer relies on e dispersive components to separate light spatially according to the wavelength, such as the diffraction grating and prism. For the non-dispersive spectrometer, light can be selected either by an optical filter or by an interferometer, such as the FT-Raman spectrometer.

#### 3.2.1. Dispersive Raman Spectrometer

The basic components of a dispersive spectrometer are the entrance slit, collimating mirror, diffraction grating, focusing mirror, and exit slit. The light enters the spectrometer through the entrance slit, passing by the collimating mirror, then the light is collimated and directed to the diffraction grating. After separation, the diffraction beam from the grating is focused to the exit slit by the focusing mirror. The two parameters of great importance to a spectrometer are wavelength range and spectral resolution, both of which are related to the dispersion property of the diffraction grating. For Raman spectroscopy applications, the wavelength range can be selected according to the excitation wavelength and the Raman shift of the target sample. High spectral resolution is preferred for purposes of resolving the weak Raman peaks.

In addition to the wavelength range and spectral resolution, the other issue of importance is the quality of the Raman spectrum measured. As discussed above, the scattering efficiency of normal Raman scattering is very low, while the intensity of Rayleigh scattering is in the order of 10^4^–10^6^ times higher than the intensity of normal Raman scattering [16]. In case the target Raman line is very close to the laser wavelength, the intensity of stray light from the Rayleigh scattering can easily exceed the intensity of Raman line and dominate its spectrum. To efficiently detect the Raman signal, the Rayleigh line should be first rejected or attenuated, and this is known as stray light rejection in a spectrometer.

**Figure 3.**
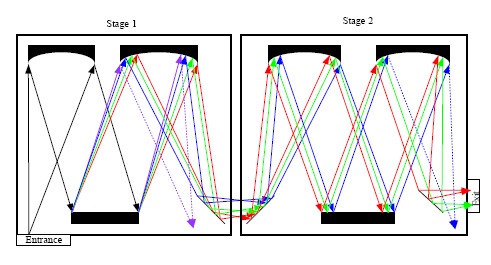
Simplified diagram of an additive double monochromator.

Both edge filters and notch filters have been used for Rayleigh light attenuation and rejection in a single stage monochromator. In addition, Rayleigh light rejection can also be achieved by using a multistage monochromator, which was widely used in earlier dispersive Raman spectrometers [26]. In case of double monochromators, the dispersion caused by the second monochromator can be added or subtracted from the dispersion of the first monochromator, so there are additive and subtractive double monochromators. Figure 3 shows the simplified diagram of an additive double monochromator.

As demonstrated in Figure 3, the rejection of Rayleigh light in a multistage monochromator is realized by using intermediate slits to block some of the unwanted wavelength band. A triple monochromator has higher Rayleigh rejection than a double monochromator. Although the multistage monochromator achieves high stray-light rejection and permits the measurement of Raman signals very close to the laser wavelength, this is compromised by the large size, complex configuration, high cost and loss of throughput. It was reported that the throughput of a triple monochromator is in the range of 3%–10%, compared to 30%–50% for a single monochromator [27].

The development of holographic notch filters and other edge filters has significantly simplified the instrumentation of a Raman spectrometer [28]. Most of the commercial spectrometers are equipped with single stage grating and filters for Rayleigh light attenuation and rejection. Compact size and low cost make the use of these spectrometers a promising technique for onsite application, and micro-spectrometers have been actively researched in academia in the past decade. Thus, double or triple monochromators are mainly limited to situations, such as in laboratory use where strong stray light rejection is required, or when the laser excitation is tuned.

Commercial Raman spectrometers are usually equipped with sophisticated but macro scale components. These spectrometers provide high spectral resolution and throughput, but are expensive. A number of efforts have been made to miniaturize the spectrometers to enable their field usage. For instance, various configurations such as single or double planar gratings [29], single mirror [30] or double mirrors have been proposed for system miniaturization. In addition, various grating designs have been investigated to optimize diffraction efficiency and spectrum resolution [31]. In past decades, different types of gratings (planar, concave, constant or varied line space gratings) were designed for micro-spectrometers.

In addition to the conventional mechanical ruling method, other advanced grating fabrication technologies have been used for gratings applied for different wavelength regions. For example, photolithography has been used to fabricate gratings with larger than 1 μm pitch, the holographic method is used for smaller pitch and aberration corrected gratings [32], and UV nano-imprint lithography [33] or deep X-ray lithography has been used to fabricate concave gratings [34]. Table 4 lists some of the micro-spectrometer designs proposed in the past decade. According to the specifications in Table 4, sub-nanometer spectral resolution is achievable in millimeter-sized spectrometers, which is important for resolving narrow Raman peaks.

#### 3.2.2. FT-Raman Spectrometer

Although the feasibility of FT-Raman was demonstrated as early as 1964 [38], due to technology limitations, the first realization of FT-Raman instrumentation was in 1986 [21]. Different from the dispersive Raman spectrometer, a FT-Raman spectrometer mainly consists of an interferometer. The measured signal is the interferogram in time domain, and the Raman spectrum can be obtained by the Fourier transformation of the interferogram.

**Figure 4.**
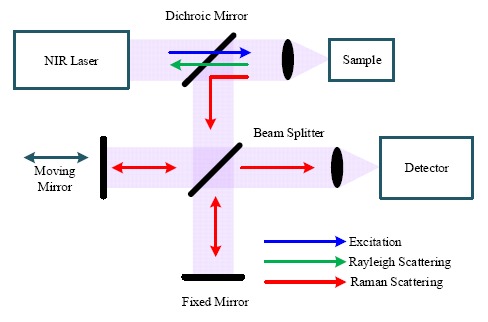
Simplified block diagram of the FT-Raman spectrometer.

Figure 4 shows a block diagram of a FT-Raman spectrometer based on a Michelson interferometer. The excitation source from a NIR laser is directed to the sample through an optical mirror and lens, which also collects the scattered signals (Rayleigh and Raman) from the sample. The dichroic mirror transmits the short wavelength Rayleigh scattering signal and reflects the longer wavelength Raman scattering signal to a beam splitter. Through the beam splitter, half of the Raman signal is transmitted to a fixed mirror and half is reflected to a moving mirror. Because of the optical path difference caused by the moving mirror, the two beams reflected from the two mirrors undergo constructive and destructive interference, determined by the travel range of the moving mirror. Finally, the signal with all frequencies is registered on the detector simultaneously and the interferogram can be obtained from the detector.

Like the dispersive Raman spectrometer, the spectral resolution is significant for a FT-Raman spectrometer. Spectral resolution (Δλ) of the FT-Raman spectrometer is determined by the maximum travel range (Δ*x*_max_) of the moving mirror [16], which can be written as

(5) $$\Delta \lambda =1/\Delta x_{\max}$$

If the maximum travel range of the moving mirror is 1 cm, then the spectral resolution of the spectrometer is 1 cm^−1^. Generally, according to the target sample, different spectral resolutions can be selected in a commercial FT-Raman spectrometer. The second parameter of importance is the wavelength range, and it is related to the type of material of the beam splitter. In addition, the cut-off wavelength of the NIR detector also determines the wavelength range, and this will be discussed later.

Two types of noise exist in the FT-Raman spectrometer, including the detector noise and the short noise of the incoming photon. In the case of detector noise limited operation, the detection mechanism improves the signal-to-noise ratio, since the interferogram contains the detector noise only once; this is known as the multiplex advantage [39]. However, under shot noise limited operation, the shot noise is equal to the square root of the mean value of the events *N*, calculated by Equation (6).

(6) $$N_{\text{Shot}}=\sqrt{N}$$

Since shot noise from all of the multiplexed wavelengths would be detected simultaneously, the shot noise from both the strong band (Rayleigh or fluorescence) and the weak Raman band is spread into the entire wavelength band. It reduces the signal-to-noise ratio of the Raman signal and gives rise to the multiplex disadvantage of the FT-Raman spectrometer. Therefore, filters are used in the FT-Raman spectrometer to attenuate the strong Rayleigh band before detection.

Compared with the dispersive Raman spectrometer, the FT-Raman spectrometer has a higher throughput, excellent frequency accuracy and precision, and higher resolution. Moreover, owing to the use of a NIR excitation source (1064 nm), fluorescence emission is efficiently suppressed. However, the use of a longer wavelength excitation source has its limitations. First of all, NIR absorption spectroscopy occurs in this region, which attenuates the incident light. Second, the Raman cross-section, which is an equivalent parameter to the absorption coefficient in IR absorption spectroscopy, is proportional to the 4th power of the incident frequency. As a consequence, Raman scattering efficiency in the longer wavelength (NIR) region is lower than that in the short wavelength (visible) region. The lower Raman scattering efficiency limits the sensitivity of the FT-Raman spectrometer, which is important in applications such as that to detect water contaminants. Therefore, FT-Raman spectrometers are mainly used when samples fluoresce, such as in forensic analysis [40] and pharmaceutical applications [41].

### 3.3. Detection

Because of the low Raman scattering efficiency, detection of the Raman signal is very challenging, and the detector should be sensitive. The detectors exploit the photoelectric effect which uses the incoming light energy to generate charge carriers that are separated and can subsequently be measured as a current at the terminals. Two key parameters associated with a detector are the quantum efficiency (QE) and the noise. QE defines the efficiency of a detector to convert optical photons to free charges and noise refers to the dark current caused by the thermal generated charge carriers. Accordingly, to observe the weak Raman signal, the detector should have high QE in the related wavelength band, low noise level and high dynamic range. To date, several types of detectors have been successfully used in Raman spectrometers, and most of them are discussed in the following subsections.

#### 3.3.1. Photomultiplier Tubes (PMT)

A PMT consists of a photocathode, series of dynodes and an anode. Photons incident on the cathode generate electrons due to the photoelectric effect. These electrons are accelerated in a high electric field between the cathode and the adjacent dynode. The accelerated electron impinges on the dynode generating additional electrons due to secondary emission. A cascade of such dynode structures quickly amplifies the number of electrons which generates a large current pulse when it impinges on the anode. Because of their high gain, PMT can detect even a single photon and thus normally works in the photon counting mode.

In comparison with other types of detectors, the advantages of PMTs are their high gain, low noise level, and short transit time. Modern PMTs have gain of above 10^5^, dark current in the range of nA, and transit time is in range of nanoseconds [42]. Their main disadvantage is that high operating voltage is required for the high gain. The operation voltage in typical commercial PMTs [43] is above 1000 V. The QE of PMTs in the visible to NIR range is below 40% [43] and is lower than commercial charge coupled detectors (CCDs). Because of their large active region (∼10 mm), PMTs are mainly used for single channel detection, and continuous wavelength measurement is realized by scanning the gratings to adjust the output wavelengths. PMTs were widely used in dispersive Raman spectrometers before the 1980s because of their high QE and low dark current. However, with the advent of high QE multichannel detectors (CCDs) in the 1980s, PMT is used less in modern Raman spectrometers.

#### 3.3.2. Charge Coupled Devices (CCDs)

**Figure 5.**
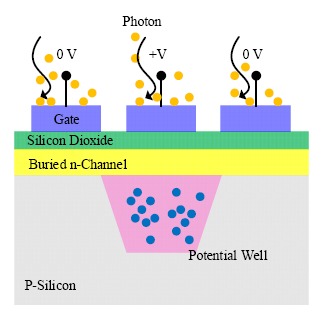
Basic structure of a charge coupled device (CCD).

The CCDs consist of a large matrix of pixel elements, the fundamental structure of which is a metal-oxide-semiconductor diode on a thin silicon substrate [44,45]. A polysilicon gate is deposited on top of each pixel, and an external bias is applied to the gate to control the potential of the region beneath the gate. By applying different biases (reverse bias and zero), an individual pixel is isolated from the neighboring pixels because of the insulating barriers (potential well shown in Figure 5). For pixels that are reverse-biased, depletion regions are formed, and charges are held and stored within the potential well up to the full well when illumination is applied. In contrast, those zero-biased pixels are transparent to the incoming photons. The number of charges generated is proportional to the intensity of the incident light flux, and the full well capacity determines the maximum light intensity that can be detected. By adjusting the bias, carriers stored in the potential well can be transferred to the output of the detector.

The active region of a CCD is ∼10 μm, which is preferred for the multichannel detection system. The multichannel detection allows the detection of multi wavelengths simultaneously. It is beneficial to reduce the integration time and the risk to damage samples if using a long time exposure. The QE of CCD is determined by the width of the depletion region and the energy of incident photons. Mainstream CCDs are silicon-based and have a peak QE of above 90% and the maximum detectable wavelength is ∼1100 nm (due to the band gap of silicon −1.12 eV). Hence, CCDs have become the mainstream detectors for commercial multichannel spectrometers (Horiba Jobin Yvon, Kyoto, Japan) in the visible region. However, the QE of most standard silicon based CCDs is limited by the available width of the depletion region which decreases rapidly beyond 900 nm.

The Raman shifts of most bacterial substances and chemicals are between 500 and 3000 cm^−1^ [46,47]. The corresponding Stokes Raman signals under 785 nm excitation are between 817 and 1026 nm, which covers the lower quantum efficiency region of standard CCDs. To measure the full Raman spectrum, novel designs have been implemented in modern advanced CCDs, by modifying either the position or width of the depletion region. Deep depletion and back illumination are the two typical techniques used in industry to increase the QE at longer wavelengths (Princeton Instruments, Andor Technology) [48]. Moreover, other techniques have also been developed to increase the detection efficiency for ultra-low light level detection, including the intensified CCD (ICCD) and Electron-Multiplying CCD (EMCCD). With respect to noise, thermal generation is the main source of the dark current [49]. Hence, cooling is the most direct and efficient way used in commercial CCDs to reduce noise [44,45,49]. Many methods such as cryogenic cooling with liquid nitrogen and thermoelectric cooling have been used.

#### 3.3.3. Silicon Avalanche Photodiode

**Figure 6.**
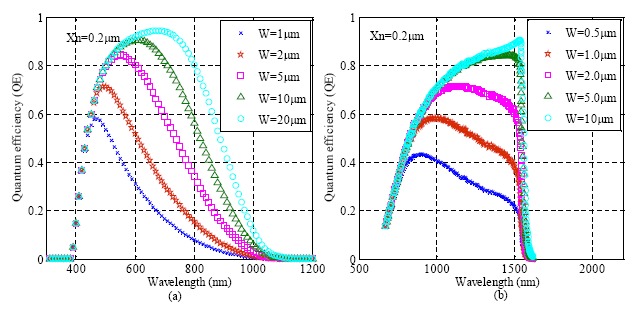
Quantum efficiency with different wavelength: (**a**) Si; (**b**) Ge. (Xn: distance from surface to depletion region; W: depletion region width).

The silicon-based avalanche photodiode (APD) is a PN junction working in the reverse-biased mode [50,51]. When incident photons are absorbed, electron-hole pairs are generated in the depletion region and multiplied through the avalanche multiplication process. APDs are highly sensitive detectors with internal gain, although their gain is lower than PMTs. Similar to CCDs, the lower QE of the photodiodes at longer wavelengths is determined by the band gap of silicon. Increasing the width of the depletion region or using a different material with a narrow band gap can improve the detection efficiency in longer wavelength. Figure 6 simulates the dependence of QE on the width of the depletion region and its distance from the surface of the detector for both silicon and germanium.

A silicon-based APD is robust, inexpensive, and easy to miniaturize. It can be operated at a lower voltage supply and is compatible with CMOS control circuitry. Although the CCD is the mainstream detector in commercial Raman instruments, APD is also a promising technology for portable and high-speed detection systems. In past decades, tremendous progress has been achieved for APDs through improvements in gain, size of active region, and response time. Commercial APDs are available from a variety of companies, for example, Hamamatsu Corporation, Boston Electronics and OSI Optoelectronics. In academia, a low noise APD with 7.6 nA/mm^2^ dark current density, fabricated by CMOS 0.35 μm technology, was reported in 2008 [52]. Another APD with a gain of 569 and 3.2 GHz 3 dB bandwidth under 10.6 V reverse bias was described in 2010 [53]. This APD was the highest gain bandwidth product among the CMOS technology fabricated APDs.

**Figure 7.**
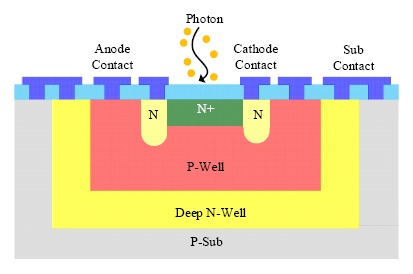
Cross section of a CMOS single photon avalanche diode (SPAD).

The single photon avalanche diode (SPAD) is essentially a PN junction biased slightly above the avalanche breakdown voltage, which is also known as the Geiger mode [54,55]. In a SPAD, single photon generated free carriers are multiplied by impact ionization in the very high-electric field of the depletion region, which then triggers the self-sustaining avalanche process. A voltage pulse can be obtained from the readout circuits, with the leading edge marking the arrival time of a photon. Owing to its single photon sensitivity, the SPAD always works in the photon counting mode. Similar to other photodetectors, the thermal generated dark count is an important issue for the application of SPADs. The past decade has witnessed the development of SPADs with higher detection efficiency, lower noise level, higher detection rate, and higher fill factor. Low cost SPADs have been realized by using inexpensive mainstream CMOS technology [54,55] (Figure 7).

The SPADs can be operated in both the free running mode and time-gated mode, which corresponds to two different types of applications using time-correlated single photon counting (TCSPC) or time-gated measurements. In the free running mode, the SPAD is always biased above its breakdown voltage until it is fired by a photon. In contrast, SPAD in the time-gated mode only performs detection in a predetermined short time window. Photons arriving out of this time window are not detected. One typical application of SPAD is time resolved fluorescence lifetime imaging [56,57].

In addition, high speed SPAD has also been tried in time-gated Raman spectroscopy. In [58], a SPAD gated in 300 ps was used to measure the Raman spectrum of olive oil. In [59], a SPAD with 1830 Hz dark count rate at 5 V excess voltage was used to detect the Raman signal of mineral willemite, and it was proved to have comparable performance to a streak camera. Although CMOS SPAD has lower QE than CCDs and PMTs, its rapid detection, single photon sensitivity, and its compatibility with CMOS control circuits makes it a promising candidate for future miniaturized Raman spectrometers.

#### 3.3.4. NIR Detectors

To detect the Raman signal in the NIR region (>1 μm), an indium gallium arsenide (InGaAs) detector is commonly used. Because of the lower bandgap, thermal generation is stronger and the InGaAs detectors are usually cooled to liquid nitrogen temperature (77 K) to control the thermally generated dark noise. However, the cut-off wavelength of the QE shifts to short wavelength with deeper cooling, and this is caused by the negative dependence of the bandgap on temperature. Take the FT-Raman spectrometer for example, when using 1064 nm laser for excitation, the maximum Raman shift of InGaAs detector drops from 3600 to 2900 cm^−1^ when the detector is cooled to 77 K [60]. In addition, the Germanium (Ge) detector is also a mature detector in the NIR region, and it possesses longer cut-off wavelength. Today, both InGaAs and Ge detectors have been used in commercial FT-Raman spectrometers.

## 4. Advanced Raman Techniques

Despite the advantages of Raman spectroscopy, using it for environmental detection of chemical contaminants is difficult due to the extremely low limit of detection (LOD) required for this application. The weak Raman spectrum compounds this challenge. To improve LOD and extend its application in the detection of low concentration samples, various techniques have been developed for Raman spectroscopy. The improvement of LOD has been realized in two ways,

(1) enhancing the scattering intensity, and
(2) reducing background noise. Raman signal has been enhanced by pre-concentrating the contaminants to enhance the Raman scattering signal. Alternatively, the background noise has been reduced by rejection of fluorescence and detector noise using special techniques.

Sample pre-concentration is the most direct strategy to enhance Raman scattering. To date, many pre-concentration techniques have been proposed for both Raman and IR absorption spectroscopy; for example, solid phase micro-extraction (SPME) [26] and isotachophoresis [61]. Microfluidic devices have been used for miniaturized pre-concentration of chemicals and contaminants for rapid and direct chemical analysis [62,63]. In [64], PDMS was used with SPME to pre-concentrate organic compounds, and the Raman signal intensity was reported to be increased by more than two orders of magnitude. However, most of the pre-concentration strategies are time consuming and the setups are complex. Alternatively, advanced techniques have been developed and applied in modern Raman instruments to either increase Raman scattering or further suppress the fluorescence background to improve the signal-to-noise ratio. Some of these advanced techniques will be discussed in the following subsections.

### 4.1. Surface Enhanced Raman Spectroscopy (SERS)

Surface enhanced Raman spectroscopy (SERS) is to date the most efficient Raman technique for very low concentration detection. Since its first observation in 1974, SERS has been broadly researched in academia and the number of papers published annually on this topic is growing rapidly. Detailed review of SERS including the fundamentals, active substrates and its application can be found elsewhere [65–67]. Herein, we will briefly discuss the development of SERS, its instrumentation and application in the detection of low concentration contaminants.

#### 4.1.1. Theory

SERS was first observed on a roughened silver electrode in 1974, and this phenomenon was explained as a consequence of increased surface area [68]. However, later researches attributed this enhancement to the combination of two mechanisms—Electromagnetic (EM) and Charge Transfer (CT) enhancements. When the incident electromagnetic wave interacts with a roughed metal substrate, the excited localized surface plasmons, oscillating perpendicular to the metal surface, can amplify the electromagnetic field near the surface. The field enhancement also amplifies the incident light which according to Equation (4), also enhances the Raman scattering intensity. In addition, in situations where the analyte is chemically bonded on to the surface, electrons can transfer between metal and analyte, which can give rise to additional amplification by chemical enhancement.

In comparison with NRS, the intensity enhancement in SERS was found to be ∼10^6^ [18]. However, if combined with a Resonance Raman scattering, even higher enhancement can be achieved (10^8^–10^9^), and enhancement of up to 10^14^ has been obtained [18]. The signal enhancement significantly improves the LOD, and the advent of single molecule SERS in the 1990s has made SERS a promising technique for environmental applications [69,70].

#### 4.1.2. SERS Substrate and Fabrication Techniques

Although high enhancement factor can be obtained in SERS, this is dependent on a variety of factors, such as the wavelength, substrate profile, and distance [65]. The greatest enhancement is usually observed for specialized substrate and for molecules absorbed on the surface of the substrate. As a consequence, available SERS substrates, or substrate fabrication techniques, play a dominant role in the performance of SERS. Early SERS substrates were electrochemically roughened electrodes. Today, a metallic nanoparticle has been overwhelmingly used in SERS instruments because of the development in nanofabrication technology, such as electron beam lithography [71] and nanosphere lithography [65].

Advantages of nanofabrication are the flexibilities to control size, shape and orientation of nanoparticles, which are of great importance to the intensity enhancement. As mentioned in [66], the wavelength of peak surface plasmon resonance varies with different nanoparticle sizes. Moreover, the type of metal substrates would also affect the enhancement. Silver has been the most commonly used metal for its ability to excite intensive plasmon resonance at visible wavelength, followed by gold [72] and copper. Accordingly, to achieve maximum enhancement for a specific wavelength band, the size of the nanoparticles should be optimized based on the excitation wavelength and available metal substrate.

#### 4.1.3. SERS in Environmental Application

The advantage of SERS in environmental application is the achievable LOD. To be applied in water monitoring, LOD of SERS should be lower than the MCL set by EPA. For example, the MCL for cyanide is 200 ppb and MCL for Arsenic is 10 ppb. Table 5 lists some of the applications of SERS in water contaminant detection. According to the results in Table 5, ultra-low concentration detection is achievable (∼ppb), in particular if combined with microfluidic preconcentration devices [73,74].

### 4.2. Time-Gated Raman Spectroscopy

In addition to enhance the Raman scattering as SERS, the signal-to-noise ratio can also be improved by reducing the noise. Sources of noise in Raman measurement can be either from the detector or from the incoming optical signal. As mentioned in the Raman detectors, thermal generated dark current is the major noise for CCDs and APDs, and the most efficient way to reduce the dark current is to cool the detector during measurement.

With respect to the noise from the incoming photons, the noise can be from both Rayleigh scattering and fluorescence emission. Since Raman scattering and Rayleigh scattering differ in wavelength, so the influence of Rayleigh scattering can be removed by using an optical filter. In contrast, the fluorescence emission band overlaps with the Raman peak for certain excitation wavelengths, which blur the Raman peaks. Overall, reducing background fluorescence and detector noise are of great importance for a high signal-to-noise ratio.

**Figure 8.**
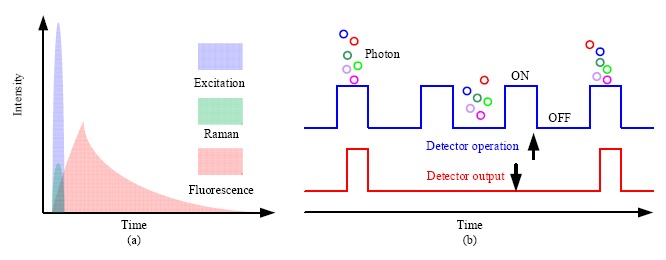
(**a**) Temporal variation of excitation, Raman scattering and fluorescence emission; (**b**) Time-gated operation of detector.

Considering the temporal distribution, Raman scattering is an instantaneous response to the excitation source, while fluorescence is emitted with a temporal distribution characterized by the so called fluorescence lifetime. The fluorescence lifetime varies from hundreds of picoseconds to tenths of nanoseconds depending on the type of samples. Consequently, if a short pulsed excitation source is used, Raman scattering and fluorescence emission can be separated in time domain (Figure 8a). To realize this separation, the width of the laser pulse should be narrow, usually in the range of hundreds of picoseconds. The repetition rate is selected according to the fluorescence lifetime, which ensures that the fluorescence from the previous cycle is fully emitted and has no contribution to the next cycle.

In time-gated Raman spectroscopy, the detection should be synchronized with the excitation source, and only signal overlapping with the excitation source in time domain can be detected, which is known as time-gated detection. Two techniques have been used for time-gated measurement, one of which is to modulate or time-gate the incoming optical signal, such as the introduction of the optical shutter; the other is to modulate the detector by gating the detector only in a predetermined short time window, and to turn off the detector after the time window. Figure 8b shows the time-gated operation of a detector, from which we can see that only photons arriving within the gate window can be detected.

#### 4.2.1. Kerr Gated Raman System

The Kerr gate is the most well-known optical shutter in time-gated Raman spectrometer for its fast response. A Kerr gate with picoseconds (25 ps) response and high repetition rate was proposed as early as the 1970s [89]. Later researches were focused on further reducing the response time. In 1999 [90], the Kerr gate was firstly introduced to Time Resolved Resonance Raman spectroscopy (TR^3^) with a response time of about 3 ps. This Kerr gate system consisted of two crossed polarizers, and a Kerr medium was placed between the polarizers. A gating pulse was used to gate on and off the Kerr gate, by varying the polarization orientation of light passing through the Kerr medium. Otherwise, no light could pass the Kerr gate due to the crossed polarizers.

In the Kerr-gate system, if the short gating pulse temporal overlaps with the Raman signal, then the Raman signal would be able to pass the gate with the fluorescence signal being suppressed. This setup has also been used for a variety of applications including plant auto-fluorescence [91], depth profiling spectra through the prostate gland and the bladder [92], and depth profiling of calcifications in breast tissue [93].

Currently, the Kerr gate has become a very popular optical shutter in time-gated Raman detection. However, owing to its fast response, the Kerr gate has been mainly used in time-resolved Raman spectroscopy which aims at analyzing the dynamic response of biological samples. For environmental application, the fast response provided by the Kerr gate is sufficient to perform the function of fluorescence rejection, but the complex setup has limited the Kerr gate to laboratory use.

#### 4.2.2. Fast Time-Gated Raman Systems

In addition to the optical shutter, fast gated detectors have also been employed in Raman spectrometers for fluorescence rejection. The most commonly used detector in the time gated Raman system is the intensified CCD (ICCD). Different from the normal CCD and the EMCCD (electron-multiplying CCD), the ICCD can be operated in the time-gated mode and perform ultra-sensitive detection down to a single photon. In an ICCD, a gain voltage controlled image intensifier tube is positioned in front of the CCD, and incident photons are multiplied inside the intensifier before being focused into a CCD. The gain voltage not only determines the multiplication, but also can gate on and off an ICCD. The ICCD has been used as an alternative technique to the Kerr gate system. Although not as fast as the Kerr gate system, most of the modern ICCDs can achieve hundreds picoseconds gating width, which is adequate for normal Raman spectroscopy [94–96].

In addition to the ICCD, other detectors have also been tried in time-gated Raman signal detection system, including the PMTs [97] and the recently developed SPADs [58,59]. SPAD can be operated in the time-gated mode by simply adjusting the supply voltage. In addition, since a SPAD is operated in the photon counting mode, then time-gated SPADs can reject both the fluorescence and the detector noise. This is because dark counts during the long gate-off time are not detected under the time-gated operation.

Compared with ICCD, SPADs have their own intrinsic gain without requiring external components such as the image intensifier. Thus, the system is more compact and compatible with CMOS control circuits. Size and CMOS compatibility are important for system miniaturization and on-line environmental sensing. In Table 6, a summary of time-gated Raman systems is presented. We note that a short gate window is achievable for both ICCD and SPAD.

## 5. Portable Raman Spectrometers for Field Applications

For purpose of field applications, a variety of portable Raman spectrometers have been developed in the industry. Comparing with benchtop Raman spectrometers, the portable Raman spectrometers are low cost, light weight, and more compact. Table 7 lists several portable Raman spectrometers and their specifications. These spectrometers can be battery powered with several hours operation time and fast acquisition can be achieved (TruScan RM and NOVA). The 785 nm laser is widely used in these instruments for general purposes of applications, and longer wavelength excitation is also applied when strong fluorescence is present during measurement (Inspector 500 and CBEx™ 1064). These instruments provide wide spectrum range with ∼10 cm**^−^**^1^ spectral resolution. They can be used for raw material identification or manufacturing process material validation.

## 6. Conclusions and Future Trends

Access to safe drinking water is an enormous global problem. An important aspect of water safety is related to its harmful chemical content. Therefore, there is need for monitoring systems that track the chemicals in the water. One powerful technique for characterizing the chemical composition of water is Raman spectroscopy. This technique is particularly well-suited for measurements of liquid samples because water is a weak Raman scatterer. However, limited by the lower Raman cross-section and strong fluorescence background, complex, large size and sophisticated instruments are usually required, so many commercial Raman spectrometers are limited to laboratory use. To be used for on-line water monitoring, the Raman spectrometer should be compact, easy to use, fast, and most importantly, the limit of detection (LOD) should meet a certain level, such as the maximum contamination level (MCL) set by the US Environmental Protection Agency (EPA). However, this is hard to achieve for normal Raman spectroscopy, even though modern Raman instruments are equipped with the most advanced spectrographs (spectrum resolution and high throughput) and detectors (above 90% quantum efficiency).

The advent of advanced Raman techniques in past decades has boosted the applications of Raman spectroscopy to water quality monitoring. The signal-to-noise ratio of the Raman spectrum has been improved either by enhancing Raman scattering or by reducing the background noise. The pre-concentration method is the most direct way to increase the intensity of the Raman signal and it was shown to enhance Raman scattering by two orders of magnitude. Further, microfluidic devices and channels have been widely researched for sample preconcentration. The advantages of the microfluidic devices are their compact size and low cost, which offer significant advantages to on-line water quality monitoring.

At present, surface enhanced Raman spectroscopy (SERS) is the most efficient technique to increase the Raman cross-section to enhance Raman scattering. The active substrate plays the dominant role in its enhancement factor, such as the type of noble metals used, particle size and orientation. The development in nanofabrication techniques benefits SERS with more functional activate substrates which facilitate the application of SERS to very low concentration detection. Combining SERS with Resonance Raman spectroscopy can result in more than ten orders of magnitude enhancement of SERS, and some such combined SERS systems have achieved LODs below the MCL. Unfortunately, not all molecules can be SERS active. Although this can be improved by extra processing of the substrate, but more research is needed to make practical systems.

Time-gated Raman detection is an emerging technique well-suited for fluorescence rejection. Because of the different temporal distribution of Raman scattering and fluorescence emission, the fluorescence signal can be suppressed by gating the detection, which then increases the signal-to-noise ratio. Both optical shutter and time-gated detectors have been used in time-gated Raman signal detection. The Kerr gate is the most commonly used optical shutter due to its ultra-fast gating response. This is very useful in time-resolved Raman spectroscopy, in which the dynamic response of samples is analyzed. In the case of on-line environmental applications, the complex setup of the Kerr gate system is not desirable. Although not as fast as the Kerr gate, gated intensified charge coupled devices and single photon avalanche diodes can also achieve a hundreds of picoseconds gating window, and this is adequate for normal Raman spectroscopy. Although time-gated detection is capable of improving the signal-to-noise ratio of the Raman spectrum, it is still lower than that in SERS.

Overall, the most significant issue for Raman spectroscopy in water quality monitoring arises from the detection of the weak Raman signal, in particular when the samples fluoresce. SERS is to date the most efficient Raman technique for water monitoring. The combination with a near infrared excitation source could not only enhance Raman scattering, but also suppress fluorescence emission. To extend the application of SERS, future work needs to be focused on the exploration of more functional activated substrates for the near infrared region. Alternatively, the time-gated detection method also has the potential to provide the fluorescence suppressed Raman spectrum. Compared with SERS, the main issue associated with time-gated detection is the lower signal-to-noise ratio, even if the fluorescence signal is sufficiently suppressed. To improve the LODs of the time-gated Raman system, combination with pre-concentration microfluidic devices could be an effective solution.

## Figures and Tables

**Table 1.** List of water contaminants and the maximum contamination level (MCL) set by the US Environmental Protection Agency (EPA).

**Category**	**Contaminant**	**MCL**	**Potential Health Effect for Long Term Exposure**	**Vibrational Spectroscopy Application**
Microorganisms	Cryptosporidium	Zero	Gastrointestinal illness	Raman, FTIR
	Giardia lamblia	Zero	Gastrointestinal illness	Raman
Disinfection by-products	Chlorite	800 ppb	Anemia, nervous system effects	Raman, NIR
	Total Trihalomethanes	80 ppb	Liver, kidney or central nervous system problems, risk of cancer	Raman, MIR,
Disinfectants	Chlorine	4 ppm	Eye/nose irritation; stomach discomfort	FTIR, NIR,
	Chlorine dioxide	800 ppb	Anemia, nervous system effects	Raman, MIR
Inorganic chemicals	Cyanide	200 ppb	Nerve damage or thyroid problems	Raman
	Copper	1.3 ppm	Liver or kidney damage	NIR
	Arsenic	10 ppb	Skin damage or problems with circulatory systems	Raman, MIR, NIR
Organic chemicals	Benzene	5 ppb	Anemia; decrease in blood platelets; increased risk of cancer	Raman, MIR, NIR
	Toluene	1 ppm	Nervous system, kidney, or liver problems	Raman, MIR, NIR
	Chlorobenzene	100 ppb	Liver or kidney problems	MIR
Radionuclides	Uranium	30 μg/L	Increased risk of cancer, kidney toxicity	Raman

ppb: parts per billion; ppm: parts per million; FTIR: Fourier transform infrared; NIR: Near infrared; MIR: mid (or middle) infrared.

**Table 2.** Comparison of different optical processes.

**Technique**	**Features**	**Instrumentation**	**Applications**
Mid-IR Spectroscopy	Absorption spectroscopy Fundamental vibration mode Restriction in liquid sample	Polychromatic source, mid-IR Interferometer, filter IR detector	Pharmaceutical and agricultural applications, food science, microbial cells, clinical chemistry, material science
NIR Spectroscopy	Absorption spectroscopy Overtone and combination Chemometrics	Polychromatic source, NIR Interferometer, grating CCD, PMT	Clinical chemistry, near infrared tomography, industrial process control, water quality
Raman Spectroscopy	Scattering spectroscopy Fundamental vibration mode Low intensity	Monochromatic source, UV, visible, NIR Grating , interferometer CCD, PMT	Pharmaceuticals and cosmetics, geology and mineralogy, semiconductor materials characterization, life science, water quality
Fluorescence Spectroscopy	Emission spectroscopy Vibration mode Presence of fluorophores	Monochromatic source, UV Grating, filter PMT, APD, CCD, SPAD	Biomedical and biochemical analysis of organic samples, fluorescence lifetime imaging

CCD: charge-coupled device; PMT: photomultiplier tube; APD: avalanche photodiode; SPAD: single photon avalanche diode.

**Table 3.** Commercial lasers used in Raman spectrometers and applications.

**Excitation Source**	**Laser Types and Wavelength**	**Techniques of Raman Spectroscopy**	**Applications**
NIR source	Diode laser: 785, 830 nm Solid state laser: Nd-YAG (1064 nm), Ti-Sapphire	FT-Raman spectroscopy (RS) Normal Raman spectroscopy Surface-enhanced-RS	Biological samples Polymers General purpose
Visible source	Ion laser: He-Ne (633 nm), He-Cd (442 nm), Ar+ (488 nm, 514 nm) Solid state laser: Nd-YAG (532 nm), Ti-Sapphire	Normal Raman spectroscopy Surface enhanced -RS Time-resolved Raman spectroscopy Resonance Raman spectroscopy	Organic components Art, archeology and forensics Semiconductor, minerals General purpose
UV source	Ion laser: He-Cd (325 nm), Ar+ (244 nm, 257 nm) Solid state laser pumped dye laser: Ti-Sapphire	UV Raman spectroscopy Resonance Raman spectroscopy Time-resolved Raman spectroscopy	Protein, DNA Natural chromophores Wide bandgap semiconductors

Nd-YAG: neodymium-doped yttrium aluminium garnet; Ti: Titanium; He-Ne: Helium-neon; He-Cd: Helium cadmium; Ar: Argon; FT: Fourier transform; UV: Ultraviolet; DNA: Deoxyribonucleic acid.

**Table 4.** Micro-spectrometers with nanometer spectral resolution.

**Wavelength**	**Grating Type and Density**	**Diffraction Order**	**Size**	**Resolution**	**Throughput/NA**	**Ref.**
450–750 nm	Double Planar, 1000 g/mm	−1	3 × 3 × 11 mm^3^	3 nm	9%	[29]
420–770 nm	Planar, 1.6 μm	1	0.5 cm^3^	0.7 nm	0.22	[30]
600–700 nm	Planar, 2 μm	1	11 × 1.5×3 mm^3^	6 nm	0.05rad	[35]
400–1030 nm	Concave, 3.2–4 μm	Multi-order	11 × 6 × 5 mm^3^	2.5 nm	0.2	[32]
580–730 nm	Concave, 4 μm		*R* = 25.8 mm	0.9 nm	0.11	[34]
1475–1625 nm	Concave, 3 μm	3	*R* = 44.4 mm	1.1 nm	0.21	[36]
512–768 nm	Concave, 6 μm	2	30 × 30 × 2 mm^3^	2.8 nm		[37]

**Table 5.** Surface enhanced Raman spectroscopy (SERS) in water contaminant application.

**Sample**	**Laser, Power, Detection Time**	**SERS Substrate**	**LOD (M)**	**Ref.**
B. subtilis	750,50 mW, CCD 1 min	AgFON: 600 nm diameter	2.1 × 10^−14^	[75]
Chromate	785, 80.2 mW, CCD TE Cooled 20 s	Au/mercaptoethyl pyridinium	5 × 10^−7^	[76]
*E. coli*	514.5, 100 mW, CCD 1–2 min	Ag nanoparticle suspension	∼10^3^ cfu/mL	[77]
Uranium	785, 60 mW	Au/aminomethyl phosphonic acid 50–60 nm	8 × 10^−7^	[78]
RDX in water	785 nm, 1 mW, CCD 10 s	Au nanoparticles 90–100 nm	1 × 10^−6^	[79]
Mercaptobenzoic Acid	785 nm, 5 mW, 2 s	Ag nanostructure on polyaniline membrane	1 × 10^−12^	[80]
Crystal violet	785 nm, 2 mW, CCD 10 s	Fractal-like Au nanostructure 30–50 nm	4.3 × 10^−9^	[81]
Thrombin	632.8 nm, 0.5 mW	Au nanoparticles 56 nm	2 × 10^−11^	[82]
Arsenic	532 nm, 20 mW	Ag, Cu nanoparticles coated with poly(vinyl pyrrolidone)	1.3 × 10^−8^	[83]
Cyanide	514 nm, 20 mW, CCD 30 s	Ag colloids 35–40 nm	1.5–2 × 10^−8^	[73]
Malachite green	514 nm, 20 mW, CCD 30 s	Hydroxylamine hydrochloride- reduced Ag colloid 40 nm	2.6–5.2 × 10^−9^	[74]
Cyanide	532 nm, 10 mW, CCD 100 s	Ag nanoparticles immobilized on oxidized silicon substrates	2.7 ×10^−7^	[84]
Perchlorate	785 nm, 1.5 mW, CCD 10 s	Ag nanoparticles on functionalized silica sol-gel films	1 × 10^−6^	[85]
Polychlorinated biphenyls	532 nm, 3.09 mW, 30 s	AgFON	5 × 10^−11^	[86]
Perchlorate	785 nm, 1.5 mW, CCD 10 s	Cystamine-modified Au nanoparticles	5 × 10^−16^	[87]
Uranyl Ion	632.8 nm, 2 mW, CCD 1 s	Ag modified polypropylene filter (PPF) substrates	4 × 10^−8^	[88]

FON: Film over nanosphere. LOD: Low limit of detection

**Table 6.** Recently developed time-gated Raman systems.

**Sample**	**Source**	**Power**	**Detector**	**Detection Window**	**Ref.**
Rhodamine 6 G	532 nm, 6.4 kHz, 900 ps	3 μJ/pulse	PMT	700 ps	[97]
Explosives	532 nm, 50 ps	15 mJ	ICCD	500 ps	[95]
PMMA	398 nm, 76 MHz, 3 ps		ICCD	250 ps	[96]
Explosives	532 nm, 10 kHz, 5 ns	140 mJ/pulse	ICCD	1 us	[98]
Pyrene/toluene Phenylacetone monooxygenase	257 nm, 76 MHz, 3 ps 405 nm, 76 MHz, 3 ps	2 mW 10 mW	ICCD	300 ps	[94]
Willemite	532 nm, 40 kHz, 500 ps	1 μJ/pulse	SPAD	33 ns	[59]
Olive oil	532 nm, 100 kHz	40 W	SPAD	300 ps	[58]

**Table 7.** Commercially developed portable Raman spectrometers.

**Portable Raman**	**Excitation Source**	**Spectral Resolution**	**Spectrum Range (cm^−1^)**	**Exposure Time Operation Time**	**Weight Size (cm)**
Thermal Scientific TruScan RM	250 mW @785 nm	8–10.5 cm^−1^	250–2875	>12 ms >4 h	0.90 kg 20.8 × 10.7 × 4.3

SciAps Inspector-300	300 mW @785 nm	6–8 cm^−1^	175–2875	X 4 h	1.70 kg 19.1 × 17.5 × 4.3

SciAps Inspector-500	300 mW @1030 nm	8–10 cm^−1^	100–2500	X 4 h	1.70 kg 19.1 × 17.5 × 4.3

SnRI CBEx™ 1064	300–400 mW @1064 nm	X	400–2300	X 4 h	11.4 × 7.9 × 5.7

Rigaku FirstGuard	10–60 mW @532 nm	10–15 cm^−1^	200–3000	20 ms–30 s 3 h	2.30 kg 12.2 × 31.1 × 31.4
	30–490 mW @785 nm	7–10 cm^−1^	200–2000	20 ms–30 s 3 h	
	30–490 mW @1064 nm	15–18 cm^−1^	200–2000	20 ms–10 s 3 h	
Wasatch Photonics NOVA	100 mW @785 nm	12.8 cm^−1^	200–2500	<1 s >5 h	0.82 kg 18.3 × 13.3 × 3.7

BWTEK NanoRAM	300 mW @785 nm	9 cm^−1^ @912 nm	176–2900	X >4 h	1.2 kg 22 × 10 × 5

## References

[1] Bartram J., Balance R., Bartram J., Balance R. (2004). Water Quality Monitoring: A Practical Guide to the Design and Implementation of Freshwater Quality Studies and Monitoring Programmes.

[2] Gowen A., Tsenkova R., Bruen M., O'donnell C. (2012). Vibrational spectroscopy for analysis of water for human use and in aquatic ecosystems. Crit. Rev. Environ. Sci. Technol..

[3] Hasan J., Goldbloom-Helzner D., Ichida A., Rouse T., Gibson M. (2005). Technologies and Techniques for Early Warning Systems to Monitor and Evaluate Drinking Water Quality: A State-of-the-Art Review.

[4] Harz M., Rösch P., Popp J. (2009). Vibrational spectroscopy—A powerful tool for the rapid identification of microbial cells at the single-cell level. Cytom. Part A.

[5] US Environmental Protection Agency Drinking Water Contaminants http://water.epa.gov/drink/contaminants/#one.

[6] Lin W., Li Z. (2009). Detection and quantification of trace organic contaminants in water using the FT-IR-attenuated total reflectance technique. Anal. Chem..

[7] Pérez-Quintanilla D., Sánchez A., del Hierro I., Fajardo M., Sierra I. (2009). Preconcentration of Zn(II) in water samples using a new hybrid SBA-15-based material. J. Hazard. Mater..

[8] Reich G. (2005). Near-infrared spectroscopy and imaging: Basic principles and pharmaceutical applications. Adv. Drug Deliv. Rev..

[9] Frost V.J., Molt K. (1997). Analysis of aqueous solutions by near-infrared spectrometry (NIRS) III. Binary mixtures of inorganic salts in water. J. Mol. Struct..

[10] Sakudo A., Tsenkova R., Tei K., Onozuka T., Ikuta K., Yoshimura E., Onodera T. (2006). Comparison of the vibration mode of metals in HNO_3_ by a partial least-squares regression analysis of near-infrared spectra. Biosci. Biotechnol. Biochem..

[11] Keren S., Zavaleta C., Cheng Z., de la Zerda A., Gheysens O., Gambhir S.S. (2008). Noninvasive molecular imaging of small living subjects using Raman spectroscopy. Proc. Natl. Acad. Sci. USA.

[12] Freudiger C.W., Min W., Saar B.G., Lu S., Holtom G.R., He C., Tsai J.C., Kang J.X., Xie X.S. (2008). Label-free biomedical imaging with high sensitivity by stimulated Raman scattering microscopy. Science.

[13] Popp J., Krafft C., Mayerhöfer T. (2011). Modern Raman spectroscopy for biomedical applications. Opt. Photonik.

[14] Bradley E.B., Frenzel C.A. (1970). On the exploitation of laser Raman spectroscopy for detection and identification of molecular water pollutants. Water Res..

[15] Collette T.W., Williams T.L. (2002). The role of Raman spectroscopy in the analytical chemistry of potable water. J. Environ. Monit..

[16] McCreery R.L. (2000). Raman Spectroscopy for Chemical Analysis.

[17] Ferraro J.R. (2003). Introductory Raman Spectroscopy.

[18] Smith E., Dent G. (2005). Modern Raman Spectroscopy: A Practical Approach.

[19] Deen M.J., Thompson E.D. (1989). Design and simulated performance of a CARS spectrometer for dynamic temperature measurements using electronic heterodyning. Appl. Opt..

[20] Kogelnik H., Porto S. (1963). Continuous helium-neon red laser as a Raman source. J. Opt. Soc. Am..

[21] Hirschfeld T., Chase B. (1986). FT-Raman spectroscopy: Development and justification. Appl. Spectrosc..

[22] Fujiwara M., Hamaguchi H., Tasumi M. (1986). Measurements of spontaneous Raman scattering with Nd: YAG 1064-nm laser light. Appl. Spectrosc..

[23] Xie C., Dinno M.A., Li Y. (2002). Near-infrared Raman spectroscopy of single optically trapped biological cells. Opt. Lett..

[24] Hargreaves M.D., Page K., Munshi T., Tomsett R., Lynch G., Edwards H.G. (2008). Analysis of seized drugs using portable Raman spectroscopy in an airport environment—A proof of principle study. J. Raman Spectrosc..

[25] Li Z., Deen M.J. (2014). Towards a Portable Raman Spectrometer Using a Concave Grating a Time-gated CMOS SPAD. Optics Express..

[26] Lewis I.R., Edwards H., Lewis I.R., Edwards H. (2001). Handbook of Raman Spectroscopy: From the Research Laboratory to the Process Line.

[27] Weber W.H., Merlin R., Weber W.H., Merlin R. (2000). Raman Scattering in Materials Science.

[28] Yang B., Morris M.D., Owen H. (1991). Holographic notch filter for low-wavenumber Stokes and anti-Stokes Raman spectroscopy. Appl. Spectrosc..

[29] Grabarnik S., Wolffenbuttel R., Emadi A., Loktev M., Sokolova E., Vdovin G. (2007). Planar double-grating microspectrometer. Opt. Exp..

[30] Grabarnik S., Emadi A., Wu H., de Graaf G., Wolffenbuttel R.F. (2008). High-resolution microspectrometer with an aberration-correcting planar grating. Appl. Opt..

[31] Li Z., Deen M.J., Fang Q., Selvaganapathy P.R. (2012). Design of a flat field concave-grating-based micro-Raman spectrometer for environmental applications. Appl. Opt..

[32] Brunner R., Burkhardt M., Rudolf K., Correns N. (2008). Microspectrometer based on holographically recorded diffractive elements using supplementary holograms. Opt. Express.

[33] Chen Y., Lee Y., Chang J., Wang L.A. (2008). Fabrication of concave gratings by curved surface UV-nanoimprint lithography. J. Vac. Sci. Technol. B.

[34] Grabarnik S., Emadi A., Wu H., de Graaf G., Wolffenbuttel R. (2009). Microspectrometer with a concave grating fabricated in a MEMS technology. Proced. Chem..

[35] Grabarnik S., Emadi A., Sokolova E., Vdovin G., Wolffenbuttel R.F. (2008). Optimal implementation of a microspectrometer based on a single flat diffraction grating. Appl. Opt..

[36] Ko C., Shew B., Hsu S. (2007). Micrograting fabricated by deep X-ray lithography for optical communications. Opt. Eng..

[37] Ko C., Lee M.R. (2011). Design and fabrication of a microspectrometer based on silicon concave micrograting. Opti. Eng..

[38] Chantry G., Gebbie H. (1964). Interferometric Raman spectroscopy using infra-red excitation. Nature.

[39] Fendel S., Freis R., Schrader B. (1997). Reduction of the multiplex disadvantage in NIR FT Raman spectroscopy by the use of interference filters. J. Mol. Struct..

[40] Edwards H.G., Villar S.E.J., Jehlicka J., Munshi T. (2005). FT–Raman spectroscopic study of calcium-rich and magnesium-rich carbonate minerals. Spectrochim. Acta Part A.

[41] Vergote G., de Beer T., Vervaet C., Remon J.P., Baeyens W., Diericx N., Verpoort F. (2004). In-line monitoring of a pharmaceutical blending process using FT-Raman spectroscopy. Eur. J. Pharm. Sci..

[42] Hamamatsu Photomultiplier Tubes. http://www.hamamatsu.com/jp/en/product/category/3100/3001/index.html.

[43] Hamamatsu Photomultiplier Tube R11102, R11102–01. http://www.hamamatsu.com/resources/pdf/etd/R11102_-01_TPMH1324E02.pdf.

[44] Hardy T.D., Murowinski R.G., Deen M.J. (1998). Charge transfer efficiency in proton damaged CCD's. IEEE Trans. Nucl. Sci..

[45] Hardy T.D., Deen M.J., Murowinski R.G. (1999). Effects of radiation damage on scientific charge coupled devices. Adv. Imaging Electron Phys..

[46] Wu Q., Hamilton T., Nelson W.H., Elliott S., Sperry J.F., Wu M. (2001). UV Raman spectral intensities of *E. coli* and other bacteria excited at 228.9, 244.0, and 248.2 nm. Anal. Chem..

[47] Iliescu T., Baia M., Miclăuş V. (2004). A Raman spectroscopic study of the diclofenac sodium–β-cyclodextrin interaction. Eur. J. Pharm. Sci..

[48] Princeton Instruments Spectroscopy Cameras. http://www.princetoninstruments.com/products/speccam/PyLoN/dsheet.aspx.

[49] Murowinski R.G., Linzhuang G., Deen M.J. (1993). Effects of space radiation damage and temperature on the noise in CCDs and LDD MOS transistors. IEEE Trans. Nucl. Sci..

[50] Deen M.J., Basu P.K. (2012). Silicon Photonics - Fundamentals and Devices.

[51] Kumar S., Deen M.J. (2014). Fiber Optic Communications - Fundamentals and Applications.

[52] Pancheri L., Scandiuzzo M., Stoppa D., Betta G. (2008). Low-noise avalanche photodiode in standard 0.35-μm CMOS technology. IEEE Trans. Electron Devices.

[53] Le M., Choi W. (2010). A silicon avalanche photodetector fabricated with standard CMOS technology with over 1 THz gain-bandwidth product. Opt. Exp..

[54] Faramarzpour N., Deen M.J., Shirani S., Fang Q. (2008). Fully integrated single photon avalanche diode detector in standard CMOS 0.18 μm technology. IEEE Trans. Electron Devices.

[55] Palubiak D., El-Desouki M.M., Marinov O., Deen M.J., Q. Fang Q. (2011). High-speed, single-photon avalanche-photodiode imager for biomedical applications. IEEE Sens. J..

[56] Rae B., Griffin C., McKendry J., Girkin J., Zhang H., Gu E., Renshaw D., Charbon E., Dawson M., Henderson R. (2008). CMOS driven micro-pixel LEDs integrated with single photon avalanche diodes for time resolved fluorescence measurements. J. Phys. D.

[57] Stoppa D., Mosconi D., Pancheri L., Gonzo L. (2009). Single-photon avalanche diode CMOS sensor for time-resolved fluorescence measurements. IEEE Sens. J..

[58] Nissinen I., Nissinen J., Lansman A., Hallman L., Kilpela A., Kostamovaara J., Kogler M., Aikio M., Tenhunen J. A sub-ns time-gated CMOS single photon avalanche diode detector for Raman spectroscopy.

[59] Blacksberg J., Maruyama Y., Charbon E., Rossman G.R. (2011). Fast single-photon avalanche diode arrays for laser Raman spectroscopy. Opt. Lett..

[60] Cutler D. (1990). Fourier transform Raman instrumentation. Spectrochim. Acta Pt. A.

[61] Walker P.A., Shaver J.M., Morris M.D. (1997). Identification of cationic herbicides in deionized water, municipal tap water, and river water by capillary isotachophoresis/on-line Raman spectroscopy. Appl. Spectrosc..

[62] Wainright A., Williams S.J., Ciambrone G., Xue Q., Wei J., Harris D. (2002). Sample pre-concentration by isotachophoresis in microfluidic devices. J. Chromatogr. A.

[63] Lafleur J.P., Rackov A.A., McAuley S., Salin E.D. (2010). Miniaturised centrifugal solid phase extraction platforms for in-field sampling, pre-concentration and spectrometric detection of organic pollutants in aqueous samples. Talanta.

[64] Nwaneshiudu I.C., Yu Q., Schwartz D.T. (2012). Quantitative solid-phase microextraction (spme)-raman spectroscopy for the detection of trace organics in water. Appl. Spectrosc..

[65] Stiles P.L., Dieringer J.A., Shah N.C., van Duyne R.P. (2008). Surface-enhanced Raman spectroscopy. Annu. Rev. Anal. Chem..

[66] Fan M., Andrade G.F., Brolo A.G. (2011). A review on the fabrication of substrates for surface enhanced Raman spectroscopy and their applications in analytical chemistry. Anal. Chim. Acta.

[67] Halvorson R.A., Vikesland P.J. (2010). Surface-enhanced Raman spectroscopy (SERS) for environmental analyses. Environ. Sci. Technol..

[68] Fleischmann M., Hendra P., McQuillan A. (1974). Raman spectra of pyridine adsorbed at a silver electrode. Chem. Phys. Lett..

[69] Kneipp K., Wang Y., Kneipp H., Perelman L.T., Itzkan I., Dasari R.R., Feld M.S. (1997). Single molecule detection using surface-enhanced Raman scattering (SERS). Phys. Rev. Lett..

[70] Nie S., Emory S.R. (1997). Probing single molecules and single nanoparticles by surface-enhanced Raman scattering. Science.

[71] Abu Hatab N.A., Oran J.M., Sepaniak M.J. (2008). Surface-enhanced Raman spectroscopy substrates created via electron beam lithography and nanotransfer printing. ACS Nano.

[72] Kneipp K., Haka A.S., Kneipp H., Badizadegan K., Yoshizawa N., Boone C., Shafer-Peltier K.E., Motz J.T., Dasari R.R., Feld M.S. (2002). Surface-enhanced Raman spectroscopy in single living cells using gold nanoparticles. Appl. Spectrosc..

[73] BurmáKyong J., KyuáLee E. (2005). Ultra-sensitive trace analysis of cyanide water pollutant in a PDMS microfluidic channel using surface-enhanced Raman spectroscopy. Analyst.

[74] Lee S., Choi J., Chen L., Park B., Kyong J.B., Seong G.H., Choo J., Lee Y., Shin K., Lee E.K. (2007). Fast and sensitive trace analysis of malachite green using a surface-enhanced Raman microfluidic sensor. Anal. Chim. Acta.

[75] Zhang X., Young M.A., Lyandres O., van Duyne R.P. (2005). Rapid detection of an anthrax biomarker by surface-enhanced Raman spectroscopy. J. Am. Chem. Soc..

[76] Mosier-Boss P., Lieberman S. (2003). Detection of anions by normal Raman spectroscopy and surface-enhanced Raman spectroscopy of cationic-coated substrates. Appl. Spectrosc..

[77] Sengupta A., Mujacic M., Davis E.J. (2006). Detection of bacteria by surface-enhanced Raman spectroscopy. Anal. Bioanal. Chem..

[78] Ruan C., Luo W., Wang W., Gu B. (2007). Surface-enhanced Raman spectroscopy for uranium detection and analysis in environmental samples. Anal. Chim. Acta.

[79] Hatab N.A., Eres G., Hatzinger P.B., Gu B. (2010). Detection and analysis of cyclotrimethylenetrinitramine (RDX) in environmental samples by surface-enhanced Raman spectroscopy. J. Raman Spectrosc..

[80] Yan J., Han X., He J., Kang L., Zhang B., Du Y., Zhao H., Dong C., Wang H., Xu P. (2012). Highly sensitive surface-enhanced Raman spectroscopy (SERS) platforms based on silver nanostructures fabricated on polyaniline membrane surfaces. ACS Appl. Mater. Interfaces.

[81] He L., Kim N., Li H., Hu Z., Lin M. (2008). Use of a fractal-like gold nanostructure in surface-enhanced Raman spectroscopy for detection of selected food contaminants. J. Agric. Food Chem..

[82] Hu J., Zheng P., Jiang J., Shen G., Yu R., Liu G. (2008). Electrostatic interaction based approach to thrombin detection by surface-enhanced Raman spectroscopy. Anal. Chem..

[83] Mulvihill M., Tao A., Benjauthrit K., Arnold J., Yang P. (2008). Surface-enhanced Raman spectroscopy for trace arsenic detection in contaminated water. Angew. Chem..

[84] Tan S., Erol M., Sukhishvili S., Du H. (2008). Substrates with discretely immobilized silver nanoparticles for ultrasensitive detection of anions in water using surface-enhanced Raman scattering. Langmuir.

[85] Wang W., Gu B. (2005). New surface-enhanced Raman spectroscopy substrates via self-assembly of silver nanoparticles for perchlorate detection in water. Appl. Spectrosc..

[86] Bantz K.C., Haynes C.L. (2009). Surface-enhanced Raman scattering detection and discrimination of polychlorinated biphenyls. Vib. Spectrosc..

[87] Ruan C., Wang W., Gu B. (2006). Surface-enhanced Raman scattering for perchlorate detection using cystamine-modified gold nanoparticles. Anal. Chim. Acta.

[88] Bhandari D., Wells S.M., Retterer S.T., Sepaniak M.J. (2009). Characterization and detection of uranyl ion sorption on silver surfaces using surface enhanced Raman spectroscopy. Anal. Chem..

[89] Ippen E., Shank C. (1995). Picosecond response of a high−repetition−rate CS optical Kerr gate. Appl. Phys. Lett..

[90] Matousek P., Towrie M., Stanley A., Parker A. (1999). Efficient rejection of fluorescence from Raman spectra using picosecond Kerr gating. Appl. Spectrosc..

[91] Knorr F., Smith Z.J., Wachsmann-Hogiu S. (2010). Development of a time-gated system for Raman spectroscopy of biological samples. Opt. Express.

[92] Prieto M.C.H., Matousek P., Towrie M., Parker A.W., Wright M., Ritchie A.W., Stone N. (2005). Use of picosecond Kerr-gated Raman spectroscopy to suppress signals from both surface and deep layers in bladder and prostate tissue. J. Biomed. Opt..

[93] LouiseáRonayne K., WilliamáParker A. (2007). Depth profiling of calcifications in breast tissue using picosecond Kerr-gated Raman spectroscopy. Analyst.

[94] Efremov E.V., Buijs J.B., Gooijer C., Ariese F. (2007). Fluorescence rejection in resonance Raman spectroscopy using a picosecond-gated intensified charge-coupled device camera. Appl. Spectrosc..

[95] Fleger Y., Nagli L., Gaft M., Rosenbluh M. (2009). Narrow gated Raman and luminescence of explosives. J. Lumin..

[96] Ariese F., Meuzelaar H., Kerssens M.M., Buijs J.B., Gooijer C. (2009). Picosecond Raman spectroscopy with a fast intensified CCD camera for depth analysis of diffusely scattering media. Analyst.

[97] Sinfield J.V., Colic O., Fagerman D., Monwuba C. (2010). A low cost time-resolved Raman spectroscopic sensing system enabling fluorescence rejection. Appl. Spectrosc..

[98] Carter J.C., Angel S.M., Lawrence-Snyder M., Scaffidi J., Whipple R.E., Reynolds J.G. (2005). Standoff detection of high explosive materials at 50 meters in ambient light conditions using a small Raman instrument. Appl. Spectrosc..

